# Development and immunity-related microRNAs of the lepidopteran model host *Galleria mellonella*

**DOI:** 10.1186/1471-2164-15-705

**Published:** 2014-08-23

**Authors:** Krishnendu Mukherjee, Andreas Vilcinskas

**Affiliations:** Department of Bioresources, Fraunhofer Institute for Molecular Biology and Applied Ecology, Winchester Str. 2, 35395 Giessen, Germany; Institute of Phytopathology and Applied Zoology, Justus-Liebig University of Giessen, Heinrich-Buff-Ring 26-32, 35392 Giessen, Germany

**Keywords:** MicroRNA, MicroRNA target prediction, Development, Metamorphosis, Immunity, Trans-generational immune priming, *Galleria mellonella*, *Metarhizium anisopliae*

## Abstract

**Background:**

MicroRNAs (miRNAs) are small non-coding RNAs that act as key players in the post-transcriptional regulation of protein synthesis. Although little is known about their role in complex physiological processes such as development and immunity, our knowledge is expanding rapidly, thanks to the use of model systems. The larvae of the greater wax moth *Galleria mellonella* are now established as model hosts for pathogens that infect insects or humans. To build on our previously-reported comprehensive *G. mellonella* transcriptome, here we describe the identification and analysis of development and immunity-related miRNAs, thus providing valuable additional data to promote the use of this model host for the analysis of complex processes.

**Results:**

To screen for miRNAs that are differentially expressed in *G. mellonella* (1) during metamorphosis or (2) following infection with the entomopathogenic bacterium *Serratia entomophila* or (3) with the parasitic fungus *Metarhizium anisopliae*, we designed a microarray containing more than 2000 insect miRNA probe sequences. We identified miRNAs that were significantly expressed in pre-pupae (16), pupae (22) and last-instar larvae infected with *M. anisopliae* (1) in comparison with untreated last-instar larvae which were used as a reference. We then used our transcriptomic database to identify potential 3′ untranslated regions that form miRNA–mRNA duplexes by considering both base pair complementarity and minimum free energy hybridization. We confirmed the co-expression of selected miRNAs (such as miR-71, miR-263a and miR-263b) with their predicted target mRNAs in last-instar larvae, pre-pupae and pupae by RT-PCR. We also identified miRNAs that were expressed in response to infection with bacterial or fungal pathogens, and one miRNA that may act as a candidate mediator of trans-generational immune priming.

**Conclusions:**

This is the first study to identify miRNAs that are predicted to regulate genes expressed during metamorphosis or in response to infection in the lepidopteran model host *G. mellonella*.

**Electronic supplementary material:**

The online version of this article (doi:10.1186/1471-2164-15-705) contains supplementary material, which is available to authorized users.

## Background

MicroRNAs (miRNAs) are small non-coding RNAs (~18–24 nucleotides in length) that can downregulate protein synthesis at the post-transcriptional level by generally base-pairing with the untranslated regions (UTRs) including, but not limited to, the 3′ UTRs of corresponding target messenger RNAs (mRNAs) [1]. Thousands of miRNAs have been identified or predicted in eukaryotes and their viruses since the first miRNA was shown to regulate development in the nematode *Caenorhabditis elegans* [2]. The first evidence that miRNAs play a key role in insect metamorphosis was reported in 2009, based on the inhibition of metamorphosis in the cockroach *Blattella germanica* by using RNA interference (RNAi) to silence the dicer-1 ribonuclease, which is known to transform pre-miRNAs into mature miRNAs [3]. The identification and functional characterization of miRNAs is an emerging discipline in biological research, but the consequences of disrupting miRNA expression are difficult to predict because individual miRNAs can ultimately modulate the synthesis of hundreds of proteins if they target mRNAs encoding regulatory proteins such as transcription factors. Therefore, it is unsurprising that many studies provide evidence for a causal link between the altered expression of individual miRNAs and human diseases including cancer, developmental abnormalities and malfunctions of the immune system [1, 4]. Although the role of miRNAs in vertebrate immunity is well established, there are few studies addressing the immunity-related functions of miRNAs in insects, as summarized in a recommended recent review [5].

Here we screened directly for miRNAs in the greater wax moth *Galleria mellonella*, focusing on genes that are differentially expressed during development or in response to pathogens that are ingested or breach the integument. The larvae of this species have become established as a classical model host for the analysis of pathogenesis, particularly the virulence factors produced by entomopathogenic viruses, bacteria, fungi and protozoa. *G. mellonella* has been successfully used as a powerful and reliable model host for human pathogens because it offers an inexpensive and ethically acceptable alternative to mammalian hosts in preclinical research [6, 7]. Furthermore, *G. mellonella* is an efficient whole-animal high-throughput system for the *in vivo* testing of antibiotics and as a source of novel leads for the development of anti-infectives [8].

To compensate at least in part for the lack of a complete genome sequence, we have recently described a comprehensive transcriptomic database [9] that has been exploited successfully e.g. to identify genes that are induced in response to infection with *Listeria monocytogenes* [10–12]. This Gram-positive bacterium causes the food-borne disease listeriosis in humans, which often results in fatal brainstem infections leading to meningitis and meningoencephalitis [13]. Furthermore, we have introduced *G. mellonella* as a model system to investigate the role of epigenetic mechanisms that modulate insect development and immunity, e.g. the role of histone acetylation in the regulation of transcriptional reprogramming during metamorphosis and infections [14]. This mechanism exerts its effects prior to transcriptional initiation because the acetylation of histones increases DNA accessibility and promotes gene expression, whereas the removal of acetyl groups has the opposite effect. In this study, we identified *G. mellonella* miRNAs that may contribute to post-transcriptional gene regulation during metamorphosis and in response to infection. To maximize the synergy between these investigations, we isolated total RNA from *G. mellonella* at the corresponding developmental stages and following infection with the same entomopathogens such as *M. anisopliae*.

Several approaches can be used to screen for miRNAs in insects. For example, large scale Solexa sequencing was used to identify miRNAs in the lepidopteran *Bombyx mori*, which has a completed genome sequence [15]. We designed a microarray imprinted with probes representing 2064 insect miRNA sequences deposited in miRBase (http://www.mirbase.org) because we have successfully applied this microarray-based approach to identify differentially-expressed miRNAs related to systemic bacterial infections or environmental stresses such as heat or starvation in the model beetle *Tribolium castaneum* [16]. Microarrays provide a cost-efficient method for the high-throughput analysis of miRNAs, and using the same experimental approach again ensures comparability between our most recent dataset and those published in earlier reports. However, in addition to screening for differentially-expressed miRNAs, we have also predicted the corresponding target mRNAs using both empirical (RT-PCR) and theoretical (miRNA–mRNA minimum free energy (MFE) hybridization) approaches.

## Results

### Microarray analysis of *G. mellonella*miRNAs expressed during metamorphosis and entomopathogenic fungal infections

**Figure 1:**
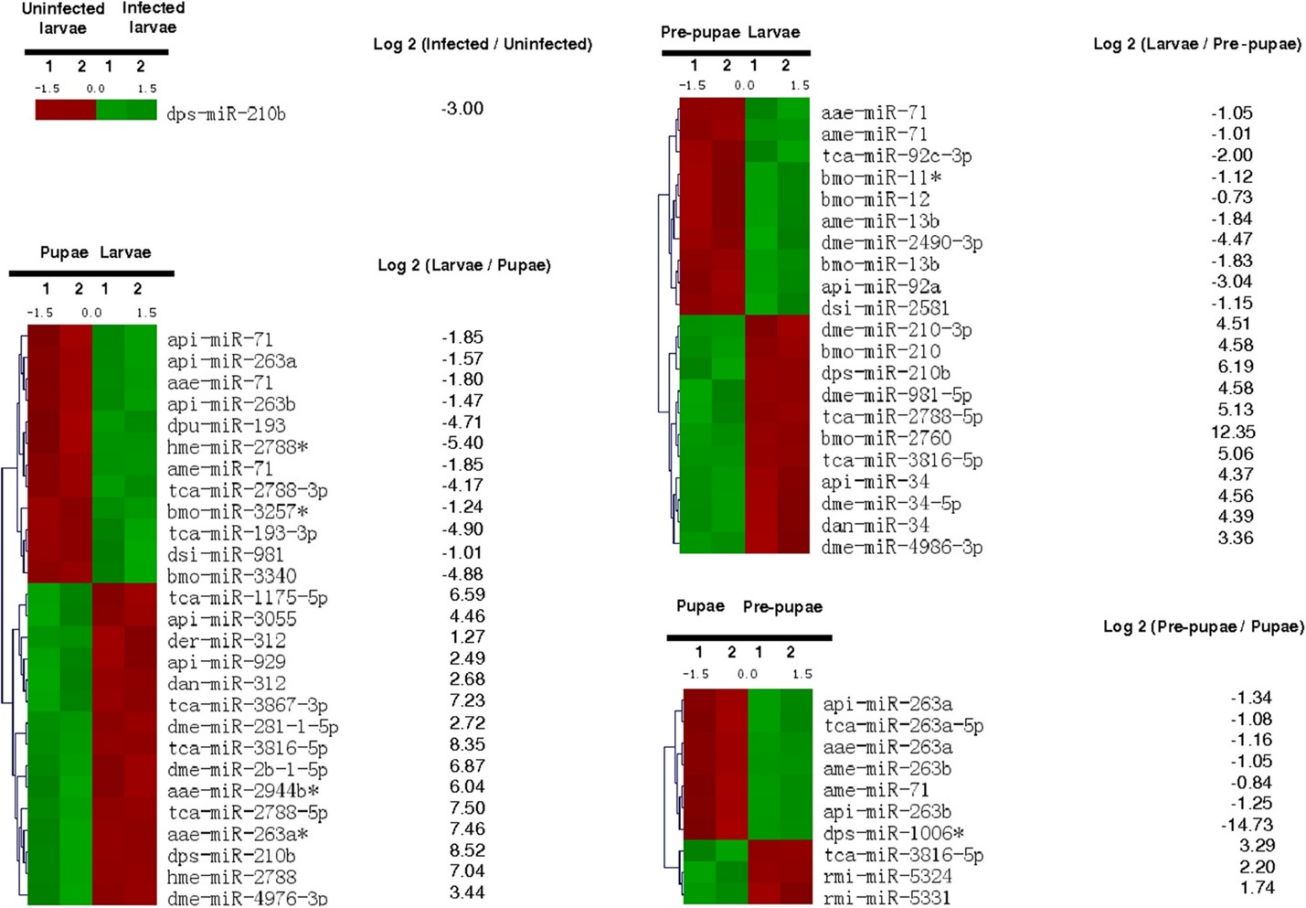
**Expression profiling of** ***G. mellonella*** **miRNAs.** The microarray heat map was generated following microarray hybridization, statistical analysis and hierarchical clustering. The heat map highlights a set of differentially-expressed miRNAs (infected vs non-infected, pre-pupae vs larvae, pupae vs larvae, and pupae vs pre-pupae. Key: red = upregulated; green = downregulated. The log score of each fold change is indicated.

**Figure 2:**
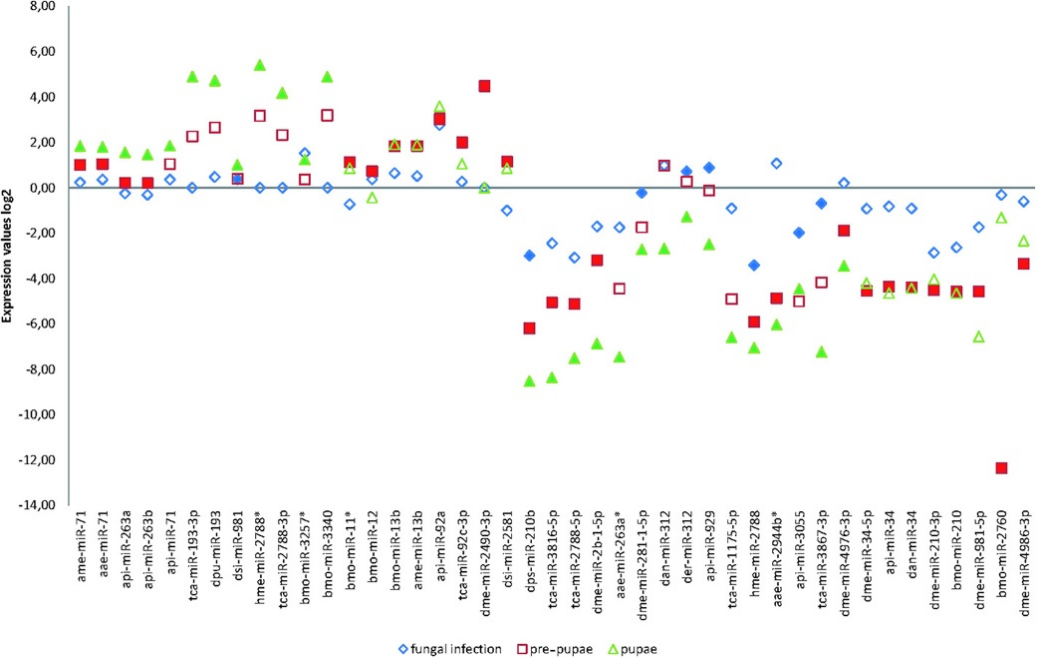
**Distribution of expressed miRNAs in pupae, pre-pupae and parasitized** ***G. mellonella*** **larvae.** The miRNAs were selected from miRBase v18 for arthropods and their expression levels were determined by microarray analysis. For the individual miRNAs presented here, the fold difference in expression was significant (p < 0.05) compared to the expression levels in untreated last-instar *G. mellonella* larvae.

We investigated miRNA expression during metamorphosis and fungal infection by designing a DNA oligonucleotide microarray containing 2064 arthropod miRNA sequences from miRBase v18. The database includes unique miRNA probes from model insects with complete genome sequences available, such as the silk worm *Bombyx mori* (559), the fruit fly *Drosophila melanogaster* (1539), the mosquito *Anopheles gambiae* (282), the red flour beetle *Tribolium castaneum* (394), the honey bee *Apis melifera* (168) and the pea aphid *Acyrthosiphon pisum* (103). All probes comprising novel and conserved mature miRNAs from different model insect species were printed in duplicate for signal verification (Figure 1). Additional information regarding sample preparation and analysis can be found in Additional file 1. The miRNA expression levels in the test samples were compared to those in untreated last-instar larvae. Taking this developmental stage as a reference, we found 1037 and 981 miRNAs (which also represent conserved miRNA sequences from insect species as duplicates) that were differentially expressed in pre-pupae and pupae, respectively. Furthermore, 1018 miRNAs were expressed in pupae relative to pre-pupae. Infection of last-instar larvae with the entomopathogenic fungus *M. anisopliae* resulted in the differential expression of 965 miRNAs. Statistical tests described in Additional file 2 were used to select 43 miRNAs in pupae, pre-pupae and infected last-instar larvae that showed significant differential expression (p < 0.05) with differences of up to 14-fold compared to naive last-instar larvae (Figure 2). We utilized miRNA sequences deposited in the miRBase database as a reference and classified among the 43 significantly differentially expressed mature miRNAs 13 as novel and 30 as conserved based on sequence homology. After removing duplicates of conserved miRNAs we identified of 16 miRNAs in pre-pupae, 21 miRNAs in pupae and 1 miRNA in *M. anisopliae* infected larvae which are differentially expressed (Additional file 3).

**Figure 3:**
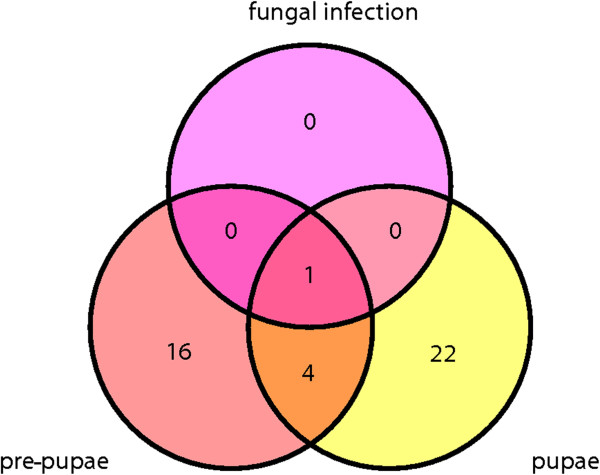
**Venn diagram showing the differential expression of miRNAs in** ***G. mellonella*** **pupae, pre-pupae and larvae infected with** ***M. anisopliae*** **, including the miRNAs that are unique to individual to or shared among particular sample types.** The miRNA sequences were sourced from miRBase v18 for arthropods and differential expression was confirmed by microarray analysis. The fold-difference in expression level for all miRNAs presented here (compared to naïve last-instar *G. mellonella* larvae) was statistically significant (p < 0.05).

Among the 42 significantly modulated miRNAs, we found that 22 were specific for pupation, 16 were specific for pre-pupation and 4 were expressed in both the stages (Figure 3). We found that dps-miR-210b was significantly overexpressed following fungal infection and during metamorphosis. The transformation of last-instar larvae into pre-pupae and pupae ultimately resulted in the significant upregulation of 10 and 12 miRNAs, respectively, and the significant downregulation of 11 and 15, respectively (Figure 1). Seven pupae-specific miRNAs were upregulated and three were downregulated compared to the expression levels observed in pre-pupae (Additional file 3). Infection with *M. anisopliae* suppressed the expression of dps-miR-210b relative to naive last-instar larvae (Figure 1). Two-factorial ANOVA confirmed the expression of *G. mellonella* miRNAs that were specific for metamorphosis and entomopathogenic fungal infection (Additional file 4).

### MiRNA target prediction

**Figure 4:**
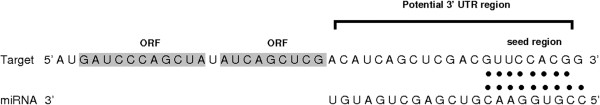
**Schematic illustration of the strategy used to predict miRNA targets.**

In the absence of a complete *G. mellonella* genome sequence, we used our comprehensive transcriptomic database to predict the putative targets of selected differentially-expressed miRNAs [9]. Nucleotide sequences at the 3′ end of individual contigs lying outside confirmed ORFs were considered to be potential 3′ UTRs, and were aligned with the mature miRNA sequences (Figure 4). This approach enabled us to determine multiple mRNA targets for at least 15 miRNAs, with gene ontologies [9] as summarized in Additional file 5: Table S1. We used an independent BLAST search to detect mRNAs in other invertebrate species that matched those we had identified in *G. mellonella*, in order to investigate the potential functional conservation of miRNAs among model insects (Additional file 6: Table S2).

**Figure 5:**
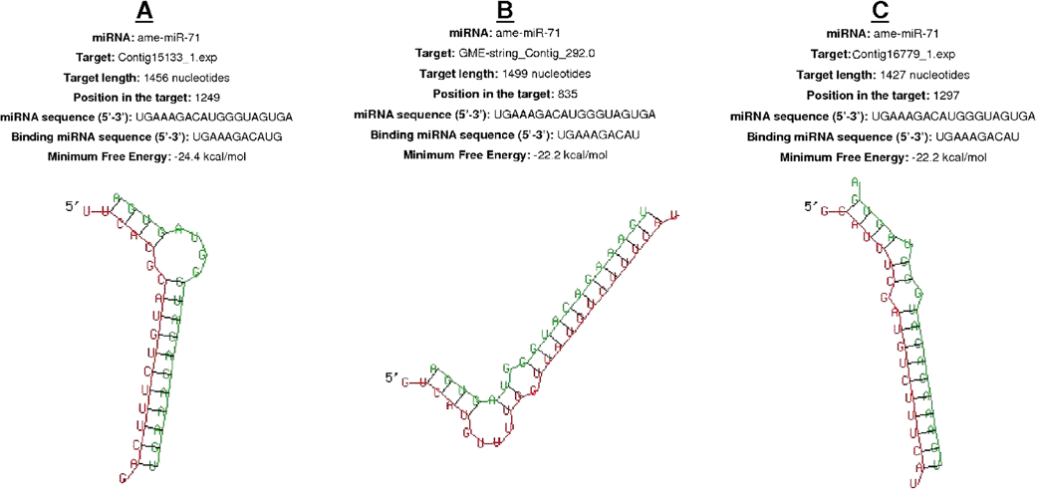
**The three best minimum free energy (MFE) duplexes formed between ame-miR-71 and the 3′-UTRs of** ***G. mellonella*** **mRNAs (the 5′ ends are marked) are shown.** The targets are **(A)** contig 15133_1.exp, **(B)** GME-string_contig_292.0 and **(C)** contig 16779_1.exp. The alignment shows the complete seed region of the miRNA hybridized to the target UTRs. Each UTR was only searched for one optimal hit.

**Figure 6:**
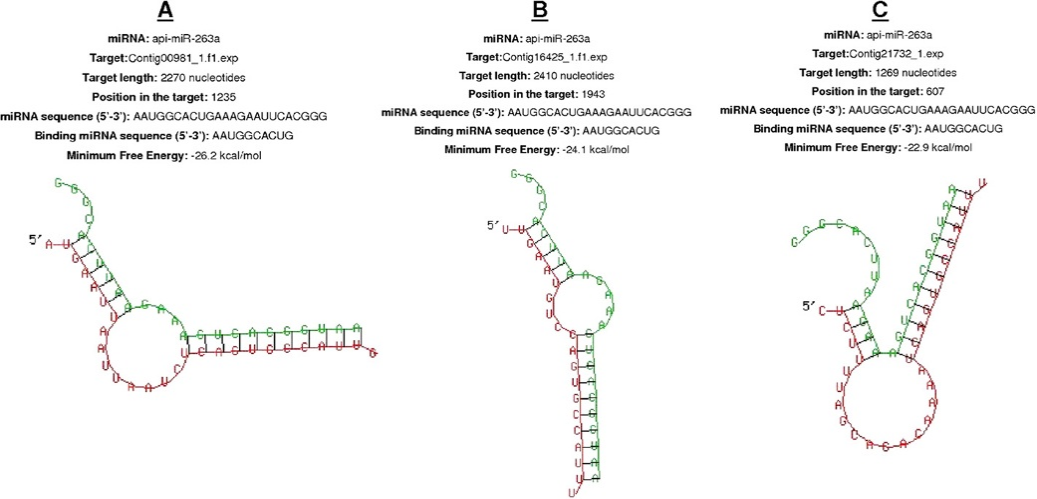
**The three best minimum free energy (MFE) duplexes formed between api-miR-263a and the 3′-UTRs of** ***G. mellonella*** **mRNAs (the 5′ ends are marked) are shown.** The targets are **(A)** contig 00981_1.f1.exp, **(B)** contig 16425_1.f1.exp and **(C)** contig 21732_1.exp. The alignment shows the complete seed region of the miRNA hybridized to the target UTRs. Each UTR was only searched for one optimal hit.

**Figure 7:**
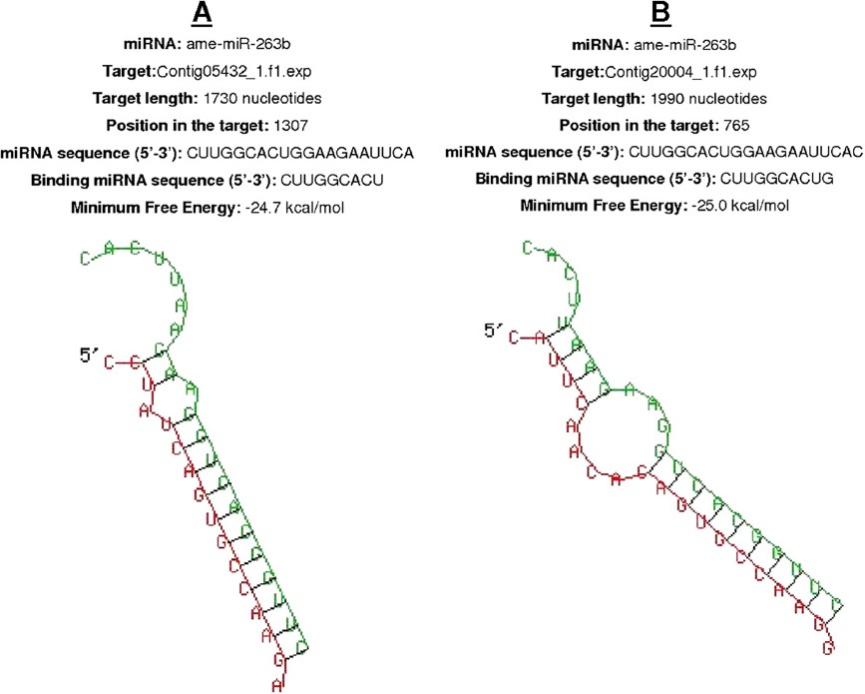
**The two best minimum free energy (MFE) duplexes formed between ame-miR-263b and the 3′-UTRs of** ***G. mellonella*** **mRNAs (the 5′ ends are marked) are shown.** The targets are **(A)** contig 05432_1.f1.exp and **(B)** contig 20004_1.f1.exp. The alignment shows the complete seed region of the miRNA hybridized to the target UTRs. Each UTR was only searched for one optimal hit.

**Figure 8:**
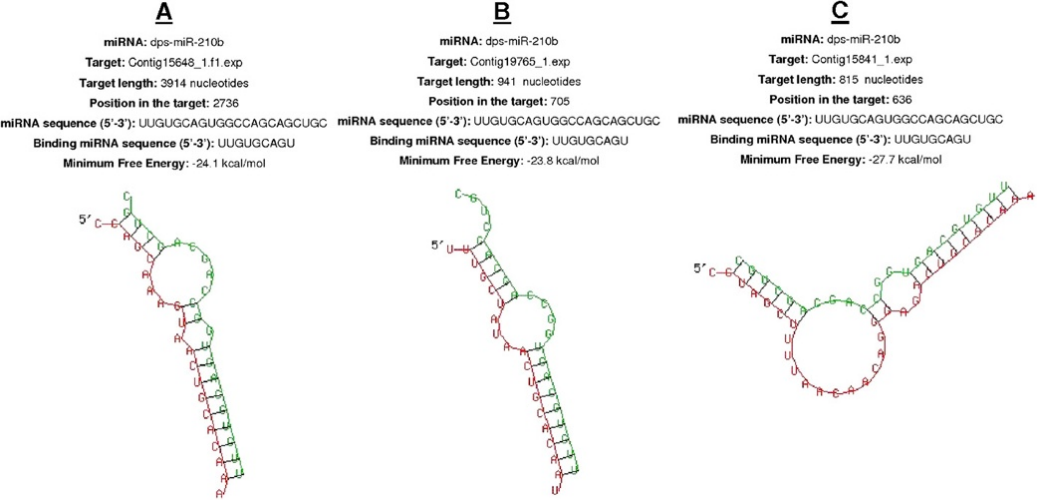
**The three best minimum free energy (MFE) duplexes formed between dps-miR-210b and the 3′-UTRs of** ***G. mellonella*** **mRNAs (the 5′ ends are marked) are shown.** The targets are **(A)** contig 15648_1.f1.exp, **(B)** contig 19765_1.exp and **(C)** contig 15841_1.exp. The alignment shows the complete seed region of the miRNA hybridized to the target UTRs. Each UTR was only searched for one optimal hit.

We validated our miRNA–mRNA target assignments using the RNAhybrid program, which predicts multiple potential binding sites for miRNAs in large target RNAs. Briefly, the program finds the energetically most favorable hybridization sites for miRNAs in a corresponding mRNA sequence, while eliminating intramolecular hybridization i.e. base pairing between target mRNA nucleotides or between miRNA nucleotides [17]. The software indicated that complete seed sequence complementarity preceded miRNA–mRNA duplex formation thus confirming the targets we identified. We found 43 miRNA–mRNA duplexes using this approach, including ame-miR-71 (Figure 5A-C), api-miR-263a (Figure 6A-C), ame-miR-263b (Figure 7A-B) and dps-miR-210b (Figure 8 A-C), shown as examples to highlight the significant overexpression during pupation, pre-pupation and fungal infection. Duplex formation by the other significantly modulated miRNAs was also confirmed utilizing the RNAhybrid software (Additional files 7– 8: Figures S1-S2).

The majority of the modulated miRNAs were found to target the transcriptional machinery, and mRNAs related to metabolism and antimicrobial responses (Additional files 5 and 6: Tables S1 and S2). For example, dps-miR-210b was downregulated by fungal infection, and targeted mRNAs encoding RNA-binding motif protein 8a, transmembrane protein 201, 1-acylglycerol-3-phosphate acyltransferase and quiescin sulfhydryl oxidase. Similarly, ame-miR-263b was specifically induced in pupae but not pre-pupae, and targeted mRNAs encoding dead box polypeptide 1 and cd27-binding protein isoform 1 (Additional file 6: Table S2).

### MiRNAs regulate the expression of target mRNAs during metamorphosis and infection

**Figure 9:**
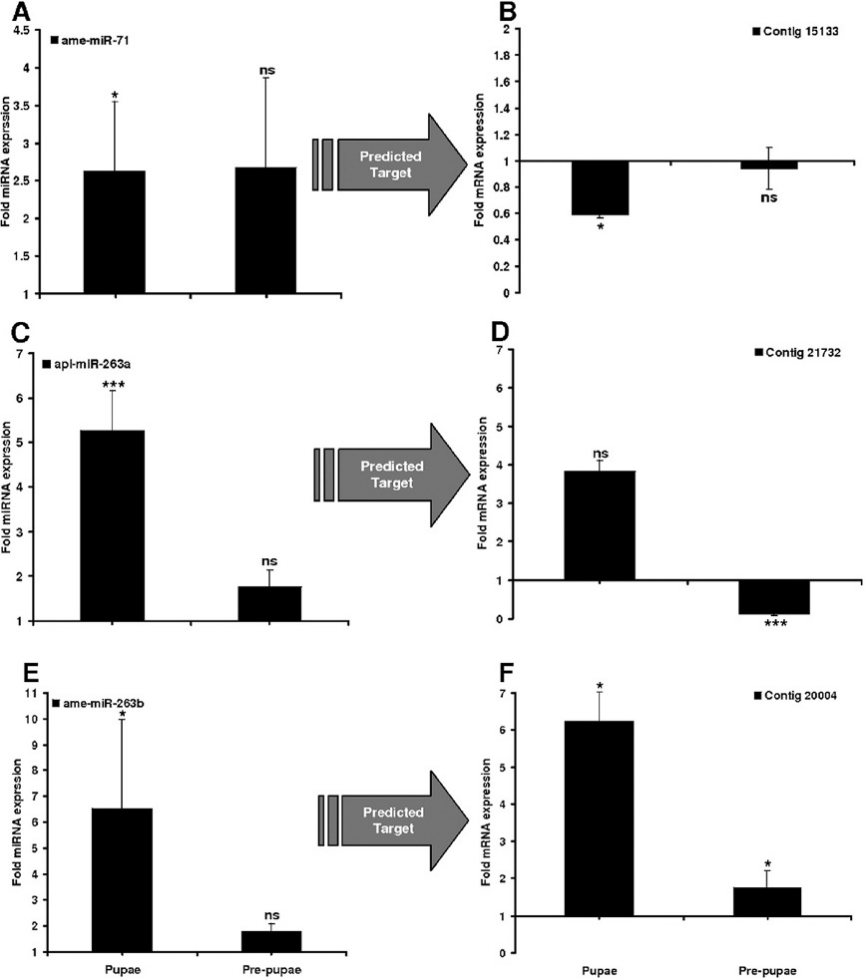
**Differential expression of miRNAs and predicted target mRNAs in** ***G. mellonella*** **pupae and pre-pupae.** The miRNAs identified by microarray analysis and their predicted mRNA targets were validated by RT-PCR in order to confirm differential expression in pupae and pre-pupae. They include **(A)** ame-miR-71 and **(B)** contig 15133; **(C)** api-miR-263a and **(D)** contig 21732; **(E)** ame-miR-263b and **(F)** contig 20004. The relative fold changes indicated for the miRNAs and mRNAs have been normalized to aae-miR-252 and 18*S* rRNA as the internal reference control. (*p < 0.05, ***p < 0.0005, ns = not significant).

**Figure 10:**
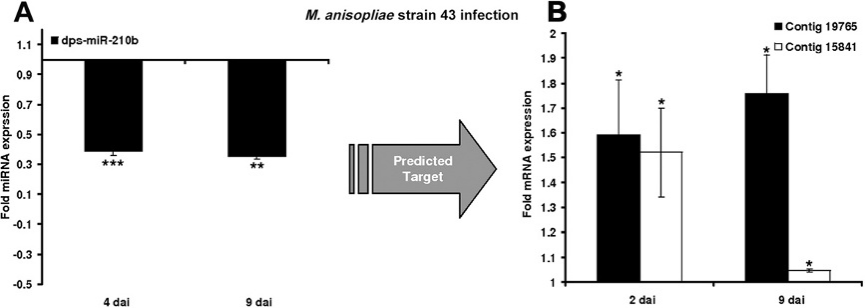
**Differential expression of miRNA and predicted target mRNAs following infection with** ***M. anisopliae*** **.** The miRNAs identified by microarray analysis and their predicted mRNA targets were validated by RT-PCR in order to confirm differential expression in infected insects. They include **(A)** dps-miR-210b and **(B)** contigs 19765 and 15841. The relative fold changes indicated for the miRNAs and mRNAs have been normalized to aae-miR-252 and 18*S* rRNA as the internal reference control. (*p < 0.05, ***p < 0.0005.

**Figure 11:**
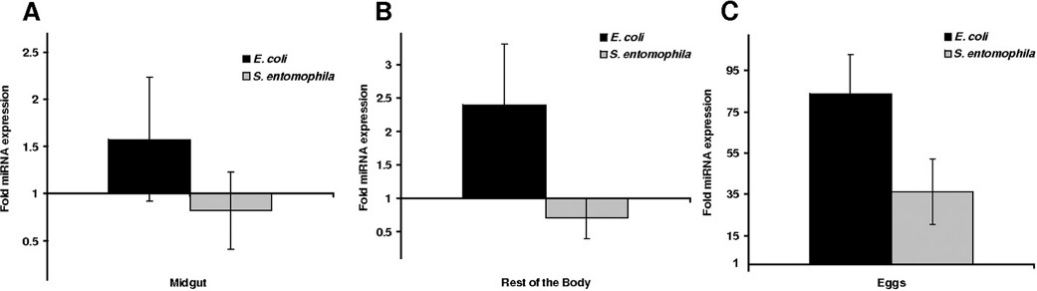
**Transgenerational expression analysis of miRNA in** ***G. mellonella*** **following exposure to bacteria contaminated diet.** Expression of api-miR-263a in midgut tissue **(A)** and rest of the body **(B)** of larvae fed either with *E. coli* or *S. entomophila.* Expression of api-miR-263a in eggs laid by female imagoes that were fed as larvae with *E. coli* or *S. entomophila* **(C)**. The transcription of this miRNA gene is given relative to that observed in larvae fed with an uncontaminated diet. Values were normalized against aae-miR-252.

We next carried out RT-PCR experiments against selected miRNAs and their predicted targets to confirm the microarray and bioinformatics results discussed above. We found that ame-miR-71 and api-miR-263a were strongly upregulated in pupae and the predicted target contigs 15133 and 21732 were strongly downregulated in pupae and pre-pupae, respectively (Figure 9A-D). In contrast, whereas ame-miR-263b was also significantly upregulated in pupae, its predicted target contig 20004 was upregulated rather than downregulated during metamorphosis (Figure 9E-F). We found that dps-miR-210b was downregulated after 4 and 9 days exposure to *M. anisopliae*, and that its predicted targets contigs 19765 and 15841 were significantly upregulated at the corresponding time points (Figure 10A-B). Additionally, we determined the expression of dme-miR-2b-1-5p, bmo-miR-13b, api-miR-34, dme-miR-981-5p, tca-miR-2788-5p and tca-miR-3867-3p in *G. mellonella* pupae and pre-pupae by RT-PCR in order to validate the microarray analysis (Additional file 9: Figure S3). Similarly, feeding larvae on a diet contaminated with *S. entomophila* resulted in the significant induction of api-miR-263a in the larval midgut, the rest of the body and even in the eggs laid by the female imagoes that were fed as larvae with entomopathogenic bacteria, whereas feeding the larvae with non-pathogenic *Escherichia coli* bacteria caused the suppression of the same miRNA (Figure 11).

## Discussion

We have carried out the first screen for miRNAs in the lepidopteran model host *G. mellonella*, using microarrays containing probes representing 2064 miRNAs from model insects with available genome sequences. We screened specifically for miRNAs that are differentially expressed during metamorphosis or in response to infection compared to the expression profile in untreated last-instar larvae. We identified several miRNAs that were significantly upregulated specifically in pre-pupae and/or pupae, indicating their potential involvement in the regulation of genes relevant to metamorphosis. We also identified miRNAs that were modulated in response to infection with the entomopathogenic fungus *M. anisopliae* or the bacterial entomopathogen *S. entomophila*.

Our recently-published comprehensive transcriptome dataset compensated, at least in part, for the absence of a complete *G. mellonella* genome sequence. The transcriptome data allowed us to identify potential 3′-UTRs and align them with the modulated miRNAs found by microarray analysis. This identified 15 miRNAs and 34 corresponding target mRNAs. The MFEs required for hybridization allowed us to interrogate the aligned duplexes to confirm the mRNA targets. We were then able to assign putative functions to the stage-specific and pathogen-induced *G. mellonella* miRNAs based on the annotated sequences of other model insects. The in silico target prediction was validated by RT-PCR to confirm the coexpression of miRNAs and their mRNA targets.

The experiments described above produced a comprehensive collection of miRNAs specifically expressed during *G. mellonella* metamorphosis. For example, we characterized development-related miRNAs such as miR-71, which targets *G. mellonella* mRNAs encoding rio kinase 1, 26S protease regulatory subunit 10b and cral trio domain-containing protein, as recently reported from the lepidopteran *Manduca sexta* [18]. Homologs of these proteins are involved in the regulation of cell cycle progression in humans. The developmental regulation of miR-71 in *G. mellonella* and *C. elegans* potentially indicates that miRNA functions are conserved, in this case by influencing germline-mediated longevity [19].

The developmental regulation of miR-263 in *G. mellonella* is interesting because it is constitutively expressed during metamorphosis in *B. mori*, and negatively regulates apoptosis, chaeta development and compound eye morphogenesis in *D. melanogaster* [20, 21]. We observed the significant induction of api-miR-263b, api-miR-263a, aae-miR-263a and ame-miR-263b in *G. mellonella* during pupation, correlating with the expression profiles in *B. mori*. Interestingly, the developmentally-modulated miRNAs we identified in *G. mellonella* were similar to those identified following the comparative analysis of pre-metamorphic and metamorphic miRNA libraries from the cockroach *B. germanica*, particularly miR-263a, miR-263b, miR-71 and miR-12, which were identified by sequencing *B. germinaca* metamorphic nymphal instars [22]. It is notable that the predicted metamorphosis-specific miRNA targets in *G. mellonella* do not include mRNAs encoding proteins involved in synthesis or binding of the ecdysone and juvenile hormones, which are known to regulate larval transformation into pupae. This suggests that miRNAs act either independently or downstream of growth hormone signaling by regulating genes that are the targets of these hormones. Similarly, miR-263a and miR-263b regulate immunity-related signal transduction by affecting the expression of genes related to the *G. mellonella* tumor necrosis factor receptor superfamily. The target mRNAs were regulated oppositionally, i.e. contig 21732 was suppressed and contig 20004 was induced during metamorphosis (Figure 9, Additional files 5 and 6: Tables S1 and S2). In addition, a recent study has shown that miR-263a influences expression of genes contributing to cellular and humoral immunity of *M. sexta* [18].

The induction of immunity-related genes is an established host response to pathogen challenge that ultimately allows the host to adapt, in a manner depending on the route of infection [23, 24]. However, little is known about the role of miRNAs in this process [25]. The injection of microbial elicitors such as bacterial peptidoglycan causes the differential expression of numerous miRNAs, whereas only a limited number are induced during natural infections [16]. Furthermore, many miRNAs induced by the experimental activation of immune responses are also upregulated following exposure to environmental stress, which makes their immunity-related functions uncertain [16]. We therefore focused on *G. mellonella* miRNAs that were expressed in response to natural infections with entomopathogens, either by ingestion (*S. entomophila*) or via the integument (*M. anisopliae*).

We previously used this strategy to address transcriptional reprogramming by histone acetylation during *G. mellonella* metamorphosis and following the penetration of the cuticle by *M. anisopliae* [14]. We provided experimental evidence that infection caused an imbalance between the enzymes responsible for histone acetylation and deacetylation, resulting in transcriptional reprogramming following infection and ultimately larval mortality. Simultaneous inhibition of the enzyme complexes using commercial inhibitors either advances or delays the transformation of *G. mellonella* larvae into pupae, the outcome depending on both transcriptional and post-transcriptional epigenetic mechanisms. We confirmed the miRNA-dependent post-transcriptional regulation of infection and development by testing the same parameters (metamorphosis and infection with entomopathogens) suggesting potential crosstalk with other epigenetic mechanisms such as histone acetylation in *G. mellonella*.

The development of immunity against entomopathogenic fungi in *G. mellonella* involves the expression of antimicrobial peptides (AMPs) [26]. A small number of miRNAs have been shown to downregulate AMP genes in insects that are naturally infected with pathogens [25]. This is also evident from our data, e.g. the significant downregulation of dps-miR-210b in response to entomopathogenic fungi. NF-κB1 is a miR-210 target, which negatively regulates the LPS-induced production of pro-inflammatory cytokines by macrophages [27]. The downregulation of dps-miR-210b in *G. mellonella* caused the significant upregulation of targets represented by contigs 19765 and 15841, which are functionally associated with transcriptional repressor activity, metabolism, inflammation, and growth regulation (Additional file 5: Table S1). This clearly reflects the parasitic behavior of *M. anisopliae* in this insect model, i.e. its ability to suppress the immune response and thus exploit host resources during the infection cycle.

In contrast to fungi which can infect insects via the exoskeleton [26], bacterial pathogens are ingested with contaminated food. The consumption of diets containing pathogenic bacteria results in the disruption of gut homeostasis, leading to dysbiosis and other gastrointestinal diseases [28]. RT-PCR experiments confirmed the expression of selected miRNAs in the midgut, rest of the body, and eggs of *G. mellonella* larvae fed on diets contaminated either with the pathogenic bacterium *S. entomophila* or a non-pathogenic strain of *E. coli*. The latter resulted in the specific upregulation of api-miR-263a whereas the same miRNA was downregulated in response to *S. entomophila*. Given that api-miR-263a regulates a number of downstream targets, the oppositional responses to different organisms suggest that distinct transcriptomic programs are orchestrated against pathogens such as *S. entomophila* and *M. anisopliae* compared to non-pathogenic organisms such as *E. coli* (Figure 11). We have recently reported that contamination of the larval diet of *G. mellonella* with *S. entomophila* results in specific immune responses both in the gut of fed larvae and in the eggs laid by females that consumed these entomopathogenic bacteria when they were larvae implicating specific trans-generational immune priming [29]. Complementarily, we report here expression of api-miR-263 both in the midgut of larvae fed with *S. entomophila* and in the eggs of females that were fed with these bacteria when they were larvae. These data implicate a role of api-miR-263 in trans-generational immune priming. However, it remains to be elucidated whether this or other miRNAs are transferred from individuals that are exposed to a pathogen to the next generation in order the mediate transgenerational immune priming.

## Conclusions

Our microarray-based screening approach identified several *G. mellonella* miRNAs that are differentially expressed during metamorphosis or in response to natural entomopathogenic infections. Putative targets were predicted for miRNAs showing the most significant modulation. These in silico predictions were then validated by quantifying the levels of miRNAs and target mRNAs by RT-PCR. We identified numerous miRNAs that may contribute to the regulation of gene expression during metamorphosis and also individual miRNAs that are modulated in response to the parasitic fungus *M. anisopliae* and the bacterial pathogen *S. entomophila.*

## Methods

### Maintenance and infection of insects

*G. mellonella* larvae were reared on an artificial diet (22% maize meal, 22% wheat germ, 11% dry yeast, 17.5% beeswax, 11% honey and 11% glycerin) at 32°C in darkness. Last-instar larvae, each weighing 250–350 mg, were used in all experiments. The transformation of larvae into pre-pupae and pupae was monitored and insects at the appropriate stages for analysis were selected randomly.

The parasitic fungus *M. anisopliae* strain 43 was obtained from the Julius-Kühn-Institute, Darmstadt, Germany and maintained on potato dextran agar (Carl Roth, Germany) at 27°C for 10 days to initiate conidiogenesis. Conidia were washed with 0.02% Triton X-100, sonicated and filtered through miracloth to remove mycelia, and isolated conidia (3000/ml) were applied topically over the cuticle of the last instar larvae to mimic a natural infection. Inoculated larvae were maintained at 27°C on the abovementioned artificial diet.

Entomopathogenic *S. entomophila* and non-pathogenic *E. coli* were obtained from DSMZ and grown aerobically in Luria broth (LB; Carl Roth, Germany) at 37°C and on LB agar plates. Overnight cultures were washed three times with 1x PBS before each culture was added to separate artificial diet preparations (~500 μl/g). Ten last-instar larvae were presented with 5 g of the contaminated diet prior to RNA isolation after 24 hours of feeding.

### Microarray analysis

For the analysis of developmentally-regulated gene expression, RNA was isolated from *G. mellonella* last-instar larvae, pre-pupae and pupae as previously described [10]. For the analysis of immunity-related genes, RNA was isolated from last-instar *G. mellonella* larvae 2, 4 and 9 days after inoculation with *M. anisopliae* strain 43. The latter samples were used to represent maximum mortality [14]. RNA was isolated from at least 10 animals per treatment for each experiment. The quantity was determined using a nanodrop spectrophotometer and the integrity was confirmed using an Agilent 2100 Bioanalyzer (Agilent Technologies). Two biological replicates were used for each sample and at each time point.

Microarray analysis was carried out by LC Sciences, USA using 2 μg total RNA samples that were extended with a 3′-polyadenylate tail using polyadenylate polymerase. An oligonucleotide tag labeled with one of two fluorescent dyes was then ligated to this tail for subsequent fluorescence detection in dual-sample experiments. The microarrays were hybridized overnight on a μParaflo microfluidic chip using a micro-circulation pump (Atactic Technologies) [30]. Each detection probe comprised a chemically-modified oligonucleotide complementary to a target miRNA (from miRBase, http://www.mirbase.org) or a control RNA, and a polyethylene glycol spacer segment to separate the coding segment from the substrate. The detection probes were generated by *in situ* synthesis using PGR (photogenerated reagent) chemistry. The hybridization melting temperatures were balanced by the chemical modification of the detection probes. Hybridization was carried out in 100 μL 6x SSPE buffer (0.90 M NaCl, 60 mM Na_2_HPO_4_, 6 mM EDTA, pH 6.8) containing 25% formamide at 34°C. After hybridization, Cy3 and Cy5 tags were circulated through the microfluidic chip for dye staining. Fluorescence images were collected using a laser scanner (GenePix 4000B, Molecular Device) and digitized using Array-Pro image analysis software (Media Cybernetics). The data were processed by first subtracting the background and then normalizing the signals using a locally-weighted regression (LOWESS) filter [31]. For the two-color experiments, the ratio of the two sets of log2 transformed and balanced signals were used to calculate the p-values of the Student’s t-test. Differential expression was judged to be significant at p < 0.05.

### MiRNA target prediction

Our transcriptomic database [9] was screened with the sequence alignment editor BioEdit to identify open reading frames (ORFs) in all contigs. The 3′ ends of the contig sequences beyond the assigned ORFs were considered as 3′ UTRs and screened for complementarity with the expressed miRNA sequences identified by microarray analysis (Figure 4). Expressed miRNAs were defined as those for which the average microarray signal was above background in at least two different pools of the same treatment group. The gene ontology of the target contigs were identified as previously described [9]. The structure of miRNA–mRNA duplexes was confirmed using the RNAhybrid tool provided by the Bielefeld Bioinformatics Server, Germany [17].

### RT-PCR analysis

Relative miRNA and mRNA expression levels were determined by RT-PCR as previously described, using the same RNA sources that were used for the microarray experiments [10]. For the analysis of miRNAs, cDNA was synthesized using the miScript II miRNA first-strand synthesis and qPCR kit (Qiagen) according to the manufacturer’s instructions. Small RNA-enriched total RNA was reverse transcribed using miScript HiSpec buffer, modified oligo-dT primers with 3′ degenerate anchors and 5′ universal tag sequence for the specific synthesis of mature miRNAs. The combination of polyadenylation and the universal tag ensures that miScript primer assays do not detect genomic DNA. Primers for the selected miRNAs were designed using the miScript miRNA product-design webpage (Qiagen). Candidate miRNA expression levels were normalized against aae-miR-252, which showed uniform expression across all samples. Real-time RT-PCR was carried out using the Biorad (CFX 96) Mx3000P (Stratagene) system, starting with a 15-min incubation at 95°C to activate the Hot Start Polymerase followed by 40 cycles at 94°C for 15 s, 55°C for 30 s and 70°C for 30 s. The following miRNA sequences were used for primer design: dps-miR-210b, 5′-UUG UGC AGU GGC CAG CAG CUG C-3′; api-miR-263a 5′-AAU GGC ACU GAA AGA AUU CAC GGG-3′; ame-miR-71 5′-UGA AAG ACA UGG GUA GUG A-3′; ame-miR-263b, 5′- CUU GGC ACU GGA AGA AUU CAC-3′; dme-miR-2b-1-5p 5′- GUC UUC AAA GUG GCA GUG ACA UG-3′; bmo-miR-13b 5′-UAU CAC AGC CAU UUU UGA CGA GU-3′; api-miR-34 5′ UGG CAG UGU GAU UAG CUG GUU-3′, dme-miR-981-5p 5′- CGG GUU UCG UUA GCA GCG GGC U-3′, tca-miR-2788-5p 5′- UGG GGU UUC UUA GCG GCA UUU-3′, and tca-miR-3867-3p 5′- UAC ACC GUU CCC GUU AUU UGC AGC GG-3′. The control miRNA sequence was aae-miR-252, 5′-UAA GUA CUA GUG CCG CAG GAG-3′.

The amplification of specific target mRNAs by RT-PCR was carried out as previously described [12] using the following primer sequences: contig19765_1.exp-fwd 5′-CTT TCG AAA TTG CGC TGA GT-3′ and -rev 5′-GTT ACT CCC GGT CGT GTG TT-3′; contig15133_1.exp-fwd 5′-CAC GCA TGT CTT TCA GTC GT-3′ and -rev 5′-GGA GCG TCC CAG ATT TTC TT-3′; contig21732_1.exp-fwd 5′-CCA GAG ATC AGG GTT TGG AG-3′ and -rev 5′-TGG CAC TGA TTT TGT CTG CT-3′; contig15841_1.exp-fwd 5′-GCT GTT TGG CTT TTT CCA AG-3′ and -rev 5′-TTC CAC GAC ACC ATA AAC CA-3′; contig20004_1.f1.exp- fwd 5′-CAT TCA ACA CAG TGC CAA GG-3′ and -rev 5′-CAG CCT GCA AGT GTT TTT CA-3′; and the housekeeping gene 18*S* rRNA-fwd 5′-ATG GTT GCA AAG CTG AAA CT-3′ and -rev 5′-TCC CGT GTT GAG TCA AAT TA-3′.

## Electronic supplementary material

Additional file 1: **Sample preparation and analysis.** (PDF 19 KB)

Additional file 2: **Sample analysis.** (PDF 13 KB)

Additional file 3: **MicroRNAs showing stage specific expression.** (XLS 56 KB)

Additional file 4: **MicroRNAs showing expression during entomopathogenic fungal infection and metamsorphosis, 2-way ANOVA with mean signal intensities (red p-values <0.0001, orange p-values <0.005, blue p-values <0.05).** (XLS 101 KB)

Additional file 5: Table S1: Gene ontology (GO) analysis of miRNA targets in *G. mellonella.* (DOC 64 KB)

Additional file 6: Table S2: Homology between selected *G. mellonella* miRNA targets and related sequences in other arthropods. (DOC 92 KB)

Additional file 7: Figure S1: The best minimum free energy (MFE) duplexes formed between (1) bmo-miR-13b, (2) api-miR-92a, (3–4, 7) tca-miR-1175-5p, (5) dsi-miR-2581, (6) bmo-miR-2760, (8–10, 13, 14) der-miR-312, (11, 12, 15, 16) api-miR-71 and the 3′-UTRs of *G. mellonella* mRNAs (the 5′ ends are marked) are shown. The targets are (1) contig 21322_1.exp, (2) contig 00597_1.f1.exp, (3) contig 00732_1.f1.exp, (4) contig 00462_1.f1.exp, (5) contig 09750_1.exp, (6) contig 01428_1.exp, (7) contig 18942_1.exp, contig 19122_1.exp, (8) contig 19122_1.f1.exp, (9) contig 02471_1.f1.exp, (10) GME-string-contig_3530.0, (11) GME-string-contig_292.0, (12) contig 15133_1.exp, (13) GME-string-contig_1146.0, (14) contig 15199_1.f1.exp, (15) contig 16779_1.exp and (16) contig 14917_2.r1.exp. The alignment shows the complete miRNAs hybridized to the target UTRs. Each UTR was only searched for one optimal hit. (TIFF 7 MB)

Additional file 8: Figure S2: The best minimum free energy (MFE) duplexes formed between (1, 13, 14) aae-miR-2944b*, (2, 7) api-miR-263b, (3) tca-miR-263a-5p, (4) aae-miR-263a, (5, 6) dme-miR-2b-1-5p, (8) aae-miR-263a , (9) dme-miR-4976-3p, (10) api-miR-929, (11) tca-miR-92a-3p, (12) aae-miR-92a, aga-miR-92a, (15) bmo-miR-92b, (16) aae-miR-13 and the 3′-UTRs of *G. mellonella* mRNAs (the 5′ ends are marked) are shown. The targets are (1) contig 02192_1.exp, (2) contig 00981_1.f1.exp, (3) contig 21732_1.exp, (4) contig 00981_1.f1.exp, (5) GME-string_contig_1995.0, (6) contig 21936_1.f1.exp, (7, 8) contig 16425_1.f1.exp, (9) contig 21905_1.f1.exp, (10) contig 03661_1.exp, (11) contig 19992_1.exp, (12) GME-string-contig_3530.0, (13) GME-string-contig_1310.0, (14) contig 02672_1.f1.exp, (15) contig 17005_1.exp, and (16) contig 21322_1.exp. The alignment shows the complete miRNAs hybridized to the target UTRs. Each UTR was only searched for one optimal hit. (TIFF 7 MB)

Additional file 9: Figure S3: Differential expression of miRNAs in *G. mellonella* pupae and pre-pupae. The miRNAs identified by microarray analysis were validated by RT-PCR in order to confirm differential expression in pupae and pre-pupae. They include dme-miR-2b-1-5p, bmo-miR-13b, api-miR-34, dme-miR-981-5p, tca-miR-2788-5p and tca-miR-3867-3p. The relative fold changes indicated for the miRNAs have been normalized to aae-miR-252 as the internal reference control. (*p < 0.05, **p < 0.005, ns = not significant). (TIFF 3 MB)

## Abbreviations

miRNA: microRNA
AMP: Antimicrobial peptide
MFE: Minimum free energy
UTR: Untranslated region.
