# Supplementary material for: Development and immunity-related microRNAs of the lepidopteran model host Galleria mellonella
Source: BMC Genomics. 2014 Aug 23;15(1):705. doi: 10.1186/1471-2164-15-705 (PMC4156658; doi:10.1186/1471-2164-15-705)
Supplement: Supplementary file 2 — Additional file 2: Sample analysis. (PDF 13 KB) [file 12864_2013_6402_MOESM2_ESM.pdf]

Multiple sample analysis involves normalization, data adjustment, t-Test, and clustering.

Normalization is carried out using a cyclic LOWESS (Locally-weighted Regression) method.[1] The normalization is to remove system related variations, such as sample amount variations, different labeling dyes, and signal gain differences of scanners so that biological variations can be faithfully revealed.

Data adjustment includes data filtering, Log2 transformation, and gene centering and normalization. The data filtering removes genes (or miRNAs) with (normalized) intensity values below a threshold value of 32 across all samples. The Log2 transformation converts intensity values into Log2 scale. Gene centering and normalization transform the Log2 values using the mean and the standard deviation of individual genes across all samples using the following formula:

Value = [(Value) – Mean(Gene)]/[Standard deviation(Gene)]

t-Test is performed between "control" and "test" sample groups.[2] T-values are calculated for each miRNA, and p-values are computed from the theoretical t-distribution. miRNAs with p-values below a critical p-value (typically 0.01) are selected for cluster analysis. The clustering is done using hierarchical method and is performed with average linkage and Euclidean distance metric.[3]

All data processes, except clustering plot, are carried out using in-house developed computer programs. The clustering plot is generated using TIGR MeV (Multiple Experimental Viewer) software from The Institute for Genomic Research.

---

1 B. M. Bolstad, R. A. Irizarry, M. Astrandand T. P. Speed, (2003) "A comparison of normalization methods for high density oligonucleotide array data based on variance and bias", Bioinformatics, 19 (2), 185-193.

2 Pan, W. 2002. A comparative review of statistical methods for discovering differentially expressed genes in replicated microarray experiments. Bioinformatics 18: 546-554.

3 Eisen, M.B., P.T. Spellman, P.O. Brown, and D. Botstein 1998. Cluster analysis and display of genome-wide expression patterns. Proceedings of the National Academy of Sciences USA 95:14863-14868.
