# Supplementary material for: Development and immunity-related microRNAs of the lepidopteran model host Galleria mellonella
Source: BMC Genomics. 2014 Aug 23;15(1):705. doi: 10.1186/1471-2164-15-705 (PMC4156658; doi:10.1186/1471-2164-15-705)
Supplement: Supplementary file 5 — Additional file 5: Table S1: Gene ontology (GO) analysis of miRNA targets in G. mellonella. (DOC 64 KB) [file 12864_2013_6402_MOESM5_ESM.doc]

| **miRNA** | **Targets** | **Gene Ontology of the Target Sequences in *Galleria mellonella*** | | |
| --- | --- | --- | --- | --- |
|  |  | **Component** | **Process** | **Function** |
| aae-miR-92a, aga-miR-92a | GME-string_Contig_3530.0 |  | proteolysis |  |
| api-miR-92a | Contig00597_1.f1.exp | extracellular region |  | chitin binding |
| ame-miR-71 | Contig15133_1.exp | protein complex, nucleus; cytosol | protein catabolic process | ATP binding, nucleoside-triphosphatase activity |
| ame-miR-71 | Contig16779_1.exp | intracellular | transport | transporter activity |
| dsi-miR-2581 | Contig09750_1.exp |  | pyrimidine ribonucleoside triphosphate biosynthetic process | kinase activity |
| bmo-miR-2760 | Contig01428_1.exp |  |  | metal ion binding; DNA binding |
| bmo-miR-2760 | Contig01617_1.exp | transcription factor TFIIF complex | positive regulation of transcription, transcription initiation from RNA polymerase II promoter, regulation of transcription from RNA polymerase II promoter | general RNA polymerase II transcription factor activity, DNA binding, transcription activator activity, transcription initiation factor activity, catalytic activity |
| api-miR-263b | Contig20004_1.f1.exp |  | signal transduction, immune response | viral receptor activity, CD27 receptor binding, tumor necrosis factor receptor binding |
| dme-miR-2b-1-5p | GME-string_Contig_1995.0 | mitochondrial proton-transporting ATP synthase complex, coupling factor F(o), lipid particle | ATP synthesis coupled proton transport | hydrogen-exporting ATPase activity, phosphorylative mechanism; F:hydrogen ion transporting ATP synthase activity, rotational mechanism; proton-transporting ATPase activity, rotational mechanism |
| der-miR-312 | Contig19122_1.f1.exp | endoplasmic reticulum membrane; microsome | electron transport | iron ion binding; heme binding |
| der-miR-312 | GME-string_Contig_3530.0 |  | proteolysis |  |
| der-miR-312 | GME-string_Contig_1146.0 | mitochondrion | cellular iron ion homeostasis |  |
| dan-miR-312 | Contig06266_1.exp |  | electron transport; purine base metabolic process | xanthine oxidase activity; FAD binding; molybdenum ion binding; iron ion binding; xanthine dehydrogenase activity; electron carrier activity; 2 iron, 2 sulfur cluster binding |
| dme-miR-4976-3p | Contig21905_1.f1.exp | integral to membrane | transport |  |
| api-miR-929 | Contig14917_2.r1.exp |  | ubiquitin cycle; regulation of protein metabolic process | small conjugating protein ligase activity |
| api-miR-929 | Contig03661_1.exp |  |  | binding |
| aae-miR-263a | Contig20004_1.f1.exp |  | signal transduction; immune response | viral receptor activity; CD27 receptor binding; tumor necrosis factor receptor binding |
| dps-miR-1006* | Contig19629_1.exp |  |  | RNA binding; nucleotide binding |
| rmi-miR-5324 | Contig02132_1.exp | snRNP U5 | mitosis; spliceosome assembly; cell division | RNA splicing factor activity, transesterification mechanism; transferase activity |
| rmi-miR-5331 | Contig03044_1.f1.exp |  | cyclin catabolic process; spindle assembly; histoblast morphogenesis; positive regulation of mitotic metaphase/anaphase transition; syncytial blastoderm mitotic cell cycle; protein amino acid phosphorylation; ; regulation of protein kinase activity; serine family amino acid metabolic process, cell division; female meiosis | cyclin-dependent protein kinase regulator activity; protein binding; cyclin-dependent protein kinase activity |

**Table S1.** Gene ontology (GO) analysis of miRNA targets in *G. mellonella.*
