# Supplementary material for: Development and immunity-related microRNAs of the lepidopteran model host Galleria mellonella
Source: BMC Genomics. 2014 Aug 23;15(1):705. doi: 10.1186/1471-2164-15-705 (PMC4156658; doi:10.1186/1471-2164-15-705)
Supplement: Supplementary file 6 — Additional file 6: Table S2: Homology between selected G. mellonella miRNA targets and related sequences in other arthropods. (DOC 92 KB) [file 12864_2013_6402_MOESM6_ESM.doc]

| **Table S2.** Homology between selected *G. mellonella* miRNA targets and related sequences in other arthropods | **miRNA** | **Target in *Galleria mellonella*** | **Homology in other insects** |
| --- | --- | --- | --- |
| Downregulated during entomopathogenic fungal infection | dps-miR-210b | RNA binding motif protein 8a | *Helicoverpa armigera* (Identity- 97%) |
| Downregulated during entomopathogenic fungal infection | dps-miR-210b | Transmembrane protein 201 | *Drosophila simulans* (Identity- 41%) |
| Downregulated during entomopathogenic fungal infection | dps-miR-210b | 1-acylglycerol-3-phosphate acyltransferase | *Heliothis virescens* (Identity- 86%), *Nasonia vitripennis* (Identity- 63%) |
| Downregulated during entomopathogenic fungal infection | dps-miR-210b | Quiescin sulfhydryl oxidase | *Apis mellifera* (Identity- 34%) |
| Downregulated during entomopathogenic fungal infection | dps-miR-210b | cg7759-isoform a | *Danaus plexippus* (Identity- 73%), *Apis mellifera* (Identity- 48%) |
| Upregulated in pre-pupae | bmo-miR-13b,ame-miR-13b,aae-miR-13 | Moricin-like peptide c3 | *Danaus plexippus* (Identity- 44%) |
| Upregulated in pre-pupae | tca-miR-92a-3p | similar to paramyosin (predicted) | *Bombyx mori*, (Identity- 96%), *Tribolium castaneum* (Identity- 64%) |
| Upregulated in pre-pupae | aae-miR-92a, aga-miR-92a | Thymus-specific serine protease | *Danaus plexippus* (Identity- 64%) |
| Upregulated in pre-pupae | api-miR-92a | AGAP004367-PA | *Tribolium castaneum* (Identity- 46%) |
| Upregulated in pre-pupae | dsi-miR-2581 | v-type atpase 116-kda subunit family protein | *Danaus plexippus* (Identity 69%) |
| Downregulated in pre-pupae | bmo-miR-2760 | general transcription factorpolypeptide 1 | *Danaus plexippus* (Identity 79%), *Megachile rotundata* (Identity 88%) |
| Downregulated in pre-pupae | bmo-miR-2760 | novel krab box and zinc-C2H2 type domain containing protein | *Danaus plexippus* (Identity- 79%)- |
| Upregulated in pre-pupae/ pupae | ame-miR-71 | 26S protease regulatory subunit s10b | *Manduca sexta* (identity 98%), *Bombyx mori* (identity 98%) |
| Upregulated in pupae | ame-miR-71 | rio kinase 1 | *Culex quinquefasciatus* (Identity 69%), *Tribolium castaneum* (Identity 69%) |
| Upregulated in pupae | ame-miR-71 | cral trio domain-containing protein | *Danaus plexippus* (Identity 71%), *Papilio xuthus* (Identity 66%) |
| Upregulated in pupae | aae-miR-71 | D-amino acid oxidase | *Papilio xuthus* (Identity-57%) |
| Upregulated in pupae | aae-miR-71 | protein tyrosine phosphatase-like (proline instead of catalytic arginine)member b | *Danaus plexippus* (Identity- 71%) |
| Upregulated in pupae | api-miR-71 | haloacid dehalogenase-like hydrolase domain containing 1a | *Danaus plexippus* (Identity- 79%) |
| Upregulated in pupae | api-miR-263a, tca-miR-263a | cg7781 cg7781-pa | *Papilio xuthus* (Identity 88%) |
| Upregulated in pupae | api-miR-263a, aae-miR-263a, tca-miR-263a-5p | cg1136 cg1136-pa | *Bombyx mori* (Identity- 69%) |
| Upregulated in pupae | api-miR-263a, tca-miR-263a-5p | solute carrier family 41 member 1 | *Danaus plexippus* (Identity- 85%) |
| Upregulated in pupae | api-miR-263b, ame-miR-263b | cd27-bindingprotein isoform 1 | *Megachile rotundata* (Identity- 34%) |
| Upregulated in pupae | api-miR-263b, ame-miR-263b | dead (asp-glu-ala-asp) box polypeptide 1 | *Bombus terrestris* (Identity- 76%) |
| Upregulated in pupae | dps-miR-1006* | RNA binding motif protein 41 | *Loxodonta africana* (Identity- 41%) |
| Downregulated in pupae | dme-miR-2b-1-5p | thap domain containing 9 | *Cydia pomonella* (Identity- 65%) |
| Downregulated in pupae | tca-miR-1175-5p | lethalg0469 cg32597-isoform b | *Papilio xuthus* (Identity- 97%), *Danaus plexippus* (Identity- 97%)- |
| Downregulated in pupae | tca-miR-1175-5p | psmd1 protein | *Danaus plexippus* (Identity- 96%), *Tribolium castaneum* (Identity- 83%) |
| Downregulated in pupae | tca-miR-1175-5p | anon-66da protein | *Danaus plexippus* (Identity-100%), *Tribolium castaneum* (Identity- 82%) |
| Downregulated in pupae | der-miR-312,  dan-miR-312 | cytochrome p450 | *Bombyx mandarina* (Identity-58%) |
| Downregulated in pupae | der-miR-312,  dan-miR-312 | coiled-coil domain containing 93 | *Tribolium castaneum* (Identity- 41%) |
| Downregulated in pupae | der-miR-312,  dan-miR-312 | thymus-specific serine protease | *Culex quinquefasciatus* (Identity- 38%) |
| Downregulated in pupae | der-miR-312,  dan-miR-312 | frataxin | *Aedes aegypti* (Identity- 59%), *Tribolium castaneum* (Identity- 65%) |
| Downregulated in pupae | der-miR-312,  dan-miR-312 | chromobox homolog 1 | *Tribolium castaneum* (Identity- 47%), *Nasonia vitripennis* (Identity- 57%) |
| Downregulated in pupae | dan-miR-312 | carboxylesterase | *Spodoptera litura* (Identity- 57%), *Helicoverpa armigera* (Identity- 57%) |
| Downregulated in pupae | dan-miR-312 | xanthine dehydrogenase | *Bombyx mori* (Identity- 73%),  *Danaus plexippus* (Identity- 69%) |
| Downregulated in pupae | dme-miR-2b-1-5p | mitochondrial f0 atp synthase d | *Papilio polytes* (Identity- 78%) |
| Downregulated in pupae | dme-miR-4976-3p | organic cation transporter | *Danaus plexippus* (Identity- 58%), *Aedes aegypti* (Identity- 34%) |
| Downregulated in pupae | api-miR-929 | ubiquitin-conjugating enzyme e2e 2 (ubc4 5yeast) | *Apis mellifera* (Identity- 97%), *Aedes aegypti* (Identity- 96%) |
| Downregulated in pupae | api-miR-929 | zbed1 protein | *Acyrthosiphon pisum* (Identity- 48%) |
| Downregulated in pupae | aae-miR-2944b* | pupal cuticle protein | *Bombyx mori* (Identity- 76%) |
| Downregulated in pupae | aae-miR-2944b* | acetyl-coenzyme a acyltransferase 2 (mitochondrial 3-oxoacyl-coenzyme a thiolase) | *Danaus plexippus* (Identity- 50%) |
| Downregulated in pupae | aae-miR-2944b* | potassium-dependent sodium-calcium exchanger | *Aedes aegypti* (Identity- 42%) |
| Downregulated in pupae | rmi-miR-5324 | bhlhzip transcription factor max bigmax | *Aedes aegypti* (Identity- 70%), *Acyrthosiphon pisum* (Identity- 72%) |
| Downregulated in pupae | rmi-miR-5324 | sugar transporter | *Bombyx mori* (Identity- 68%), *Danaus plexippus* (Identity- 69%) |
| Downregulated in pupae | rmi-miR-5324 | thioredoxin-like 4a | *Nasonia vitripennis* (Identity- 97%) |
| Downregulated in pupae | rmi-miR-5331 | cdc28 protein kinase regulatory subunit 1b | *Tribolium castaneum* (Identity- 79%), *Scylla paramamosain* (Identity- 76%) |
