# Supplementary figures and images for: Development and immunity-related microRNAs of the lepidopteran model host Galleria mellonella
Source: BMC Genomics. 2014 Aug 23;15(1):705. doi: 10.1186/1471-2164-15-705 (PMC4156658; doi:10.1186/1471-2164-15-705)

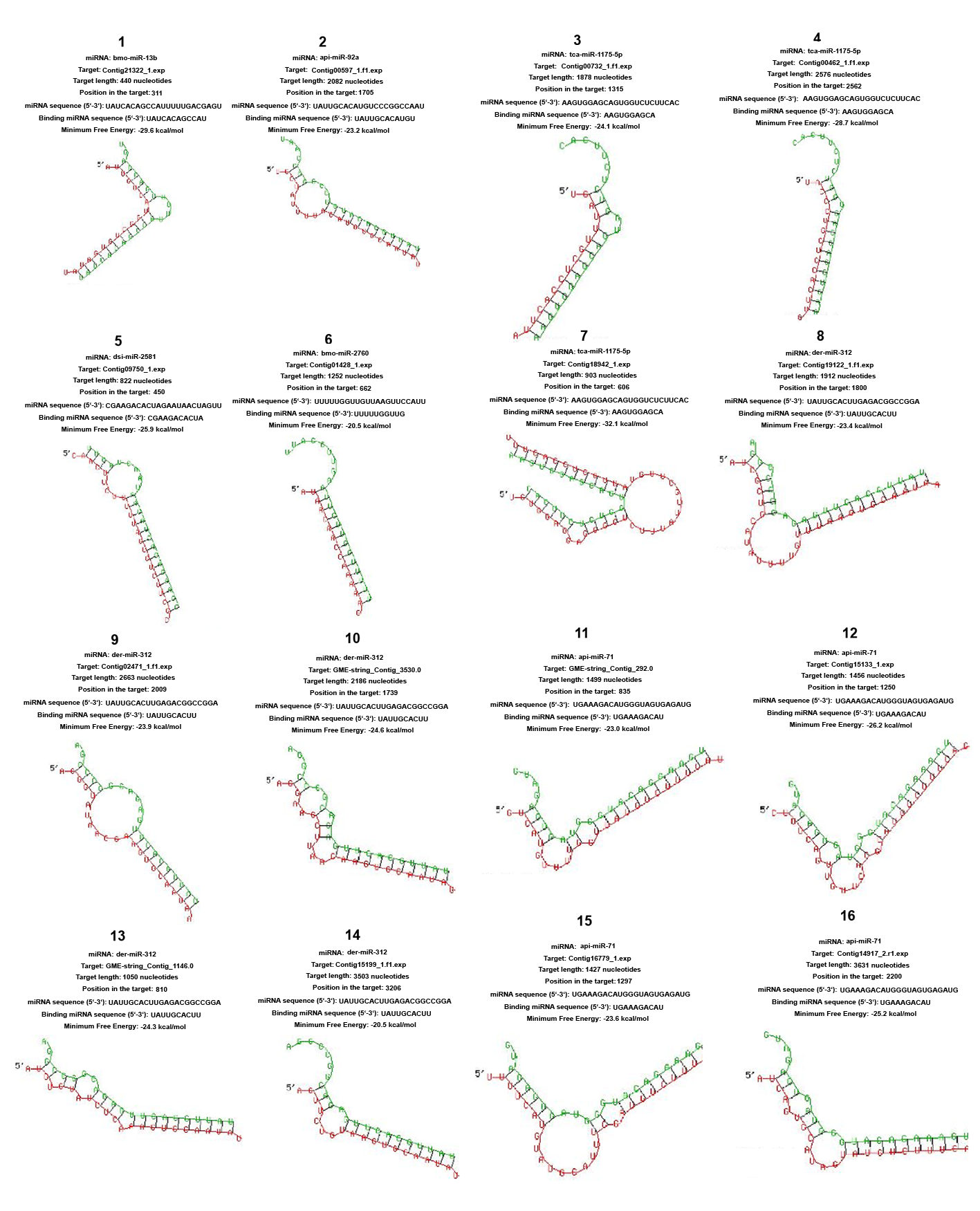

Supplement: Supplementary file 7 — Additional file 7: Figure S1: The best minimum free energy (MFE) duplexes formed between (1) bmo-miR-13b, (2) api-miR-92a, (3–4, 7) tca-miR-1175-5p, (5) dsi-miR-2581, (6) bmo-miR-2760, (8–10, 13, 14) der-miR-312, (11, 12, 15, 16) api-miR-71 and the 3′-UTRs of G. mellonella mRNAs (the 5′ ends are marked) are shown. The targets are (1) contig 21322_1.exp, (2) contig 00597_1.f1.exp, (3) contig 00732_1.f1.exp, (4) contig 00462_1.f1.exp, (5) contig 09750_1.exp, (6) contig 01428_1.exp, (7) contig 18942_1.exp, contig 19122_1.exp, (8) contig 19122_1.f1.exp, (9) contig 02471_1.f1.exp, (10) GME-string-contig_3530.0, (11) GME-string-contig_292.0, (12) contig 15133_1.exp, (13) GME-string-contig_1146.0, (14) contig 15199_1.f1.exp, (15) contig 16779_1.exp and (16) contig 14917_2.r1.exp. The alignment shows the complete miRNAs hybridized to the target UTRs. Each UTR was only searched for one optimal hit. (TIFF 7 MB) [file 12864_2013_6402_MOESM7_ESM.tiff]

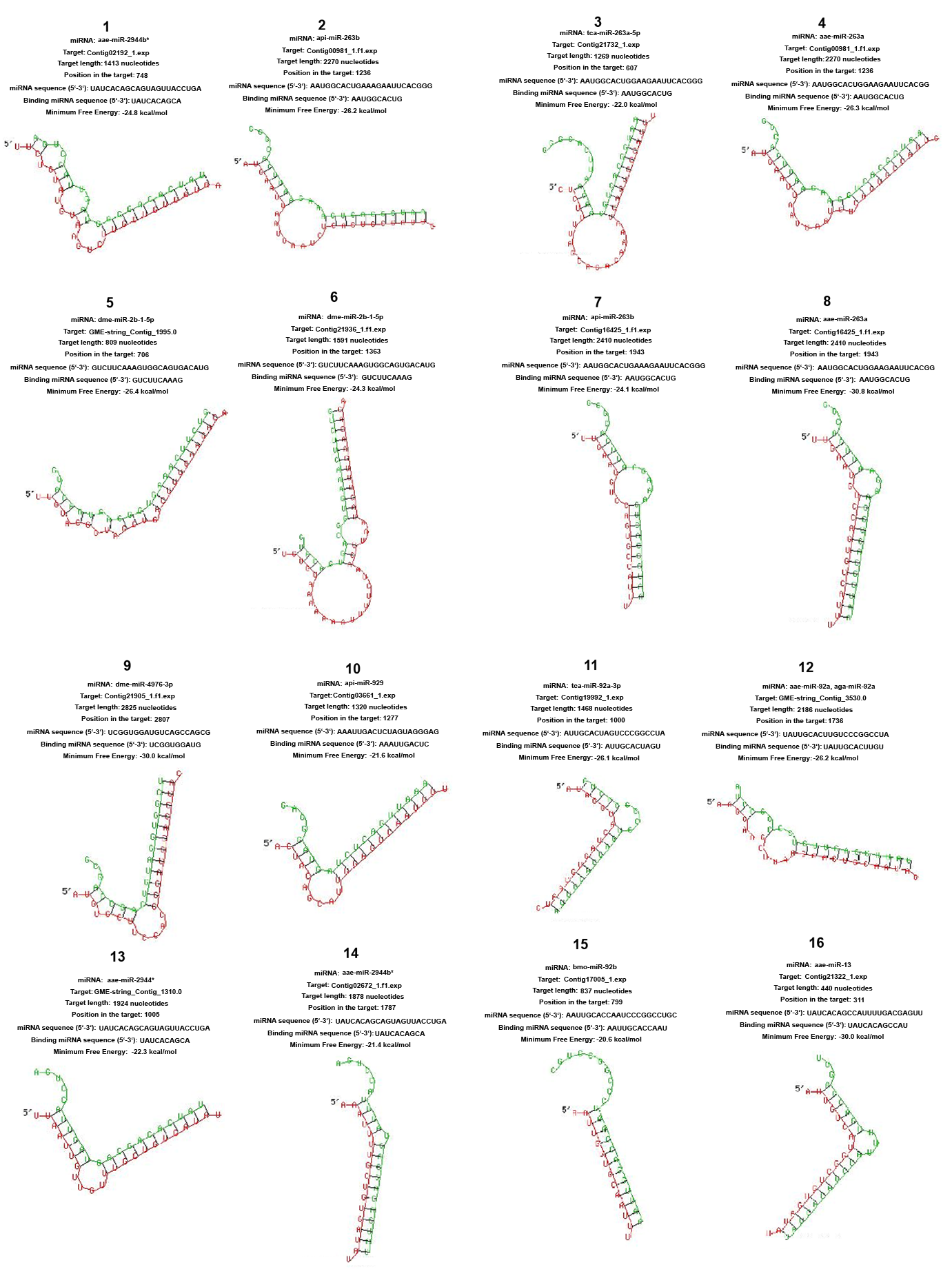

Supplement: Supplementary file 8 — Additional file 8: Figure S2: The best minimum free energy (MFE) duplexes formed between (1, 13, 14) aae-miR-2944b*, (2, 7) api-miR-263b, (3) tca-miR-263a-5p, (4) aae-miR-263a, (5, 6) dme-miR-2b-1-5p, (8) aae-miR-263a , (9) dme-miR-4976-3p, (10) api-miR-929, (11) tca-miR-92a-3p, (12) aae-miR-92a, aga-miR-92a, (15) bmo-miR-92b, (16) aae-miR-13 and the 3′-UTRs of G. mellonella mRNAs (the 5′ ends are marked) are shown. The targets are (1) contig 02192_1.exp, (2) contig 00981_1.f1.exp, (3) contig 21732_1.exp, (4) contig 00981_1.f1.exp, (5) GME-string_contig_1995.0, (6) contig 21936_1.f1.exp, (7, 8) contig 16425_1.f1.exp, (9) contig 21905_1.f1.exp, (10) contig 03661_1.exp, (11) contig 19992_1.exp, (12) GME-string-contig_3530.0, (13) GME-string-contig_1310.0, (14) contig 02672_1.f1.exp, (15) contig 17005_1.exp, and (16) contig 21322_1.exp. The alignment shows the complete miRNAs hybridized to the target UTRs. Each UTR was only searched for one optimal hit. (TIFF 7 MB) [file 12864_2013_6402_MOESM8_ESM.tiff]
